# The Role of the Pharmacist in Preventing Hepatitis B in the Context of the Opioid Crisis

**DOI:** 10.5888/pcd17.200062

**Published:** 2020-08-20

**Authors:** Catherine Freeland, Daniel J. Ventricelli

**Affiliations:** 1 Hepatitis B Foundation, Doylestown, Pennsylvania; 2 Department of Pharmacy Practice and Administration, Philadelphia College of Pharmacy, University of the Sciences, Philadelphia, Pennsylvania

Across the United States, more than 70,000 people died from drug-related overdoses in 2017, and 47,600 of those reported deaths involved an opioid (1). The largest increase in opioid-related overdoses can be attributed to a rise in the use of synthetic opioids including illicitly manufactured fentanyl, a potent opioid with rapid onset and a short duration of effect (1). Fentanyl is primarily administered by injection, and the shorter duration of effect may lead people who inject drugs to inject more frequently to stave off withdrawal symptoms between doses (2). The use of fentanyl poses an obvious risk of overdose; however, other health risks attributed to increased injection frequency are less often discussed. Increased injection frequency has been associated with a greater likelihood of syringe sharing and, in turn, increased risk of infectious disease exposure (2). The behavior has been linked to increased transmission of HIV, hepatitis B virus (HBV), and hepatitis C virus (HCV), especially when people who inject drugs do not have access to syringe service programs or other harm reduction services. A study by Lambdin and colleagues stated, “Participants reporting perceived illicit fentanyl use were more likely to report high frequency opioid use, high frequency injection and receptive syringe sharing compared with people using heroin and other street drugs but not fentanyl” (2). Because of the rise in fentanyl use, the US health care system must implement more effective strategies for reducing the risks of infectious disease transmission among people who inject drugs and broaden their focus to include HBV and other less frequently discussed infectious disease concerns associated with increased injection frequency.

Hepatitis B is transmitted through infected blood and body fluids, and common routes of transmission include unprotected sexual contact, perinatal transmission, and injection drug use (3). In the United States, up to 2.2 million individuals are chronically infected with HBV (4) and only 25.0% of adults aged 19 years or older report full vaccination coverage (5). Additionally, in 2014, populations considered to be at high risk for HBV infection reported similarly low vaccination coverage (eg, reported vaccination coverage for individuals who traveled to endemic countries [>2% prevalence of HBV infection] was 30.5%, for those who have diabetes mellitus it was 23.5%, and for those with chronic liver conditions it was 29.8%) (5).

State surveillance reports have revealed nationwide increases in acute HBV infection, with the largest increases occurring in the Appalachian region (3). An analysis of the National Notifiable Diseases Surveillance System from 2006 through 2013 assessed the incidence of acute HBV infection in 3 Appalachian states, Kentucky, Tennessee, and West Virginia, noting an increase of 114% among non-Hispanic white people aged 30 to 39 years who also reported injection drug use (3). Maine saw a 729% increase in new acute HBV cases from 2015 through 2017, with 45% of new cases coinfected with HCV (6). According to 2017 surveillance from the Centers for Disease Control and Prevention, the adjusted number of acute infections for HBV was 22,200, and there is limited data on incidence of coinfection for HBV and HCV (7). Increases in hepatitis infections have been associated with the ongoing opioid crisis and attributed to increases in injection frequency (2,3).

In the absence of effective prevention measures, the transmission and spread of viral hepatitis infections among people who inject drugs is likely to continue. Fortunately, 3 single antigen recombinant HBV vaccines (Recombivax HB [Merck], Engerix-B [GlaxoSmithKline Biologicals], and Heplisav-B [Dynavax Technologies Corporation]) and 1 combination vaccine (Twinrix [GlaxoSmithKline Biologicals]) are available and provide appropriate protection from HBV (8). All high-risk individuals are recommended to receive the full HBV vaccine series to ensure a protective antibody response, but even an incomplete series has the potential to produce clinically significant levels of protection (8). For example, the 3-dose series has been reported to produce a protective antibody response in 30% to 55% of healthy adults aged 40 years or younger following just the first dose, 75% following the second dose, and greater than 90% following the third dose of the series (8).

The HBV vaccines are safe and effective, but inconsistent access to health care services remains a barrier to obtaining the multidose immunization series among many high-risk populations. To improve vaccination coverage among people who inject drugs, new strategies should be employed to ensure the HBV vaccine series is available in a wider range of medical settings. These settings include pharmacies, primary care offices, emergency departments, social service organizations, and correctional facilities. Community pharmacists in particular have the potential to substantially increase the nation’s capacity to provide the HBV vaccine to high-risk populations and have already been called upon to play a greater role in addressing the needs of patients with opioid use disorder. Efforts by the profession include increasing pharmacy-based access to naloxone (a medication used to reverse an opioid overdose), increasing provision of sterile syringes, and ensuring medications for opioid use disorder are readily available (9). In the context of the opioid crisis, pharmacists have specifically been identified as having an important role to play in providing immunizations for health concerns associated with opioid use disorder, including HBV (10). The pharmacists’ long-standing history of involvement with vaccine storage, preparation, distribution, education, and, in more recent years, as immunization providers themselves, lends support to their ability to provide this public health service (10,11).

Today, pharmacists are recognized by the Centers for Disease Control and Prevention and the Food and Drug Administration as immunization providers (11). Some states have specific restrictions or varying laws; nevertheless, all 50 states, the District of Columbia, and Puerto Rico allow pharmacists to administer vaccinations in some capacity (11). Additionally, all accredited Doctor of Pharmacy programs across the country must provide an avenue for their graduating students to become certified in immunization delivery, thus bolstering the profession of pharmacy’s ability to provide greater immunization services with each year of new graduates (12).

Today, it is quite common for patients to receive their influenza, pneumococcal, zoster, or other single-dose vaccines from their local community pharmacy. The general convenience of community pharmacies, their extended hours compared with a traditional physician setting, and the normalization of receiving the “flu shot” from one’s local pharmacy have contributed to the successful implementation of immunization services at most community pharmacies (12). Still, this practice setting has struggled to implement models that ensure that pharmacists are able to effectively provide access to a multidose vaccine series (12). The HBV vaccine is listed among those available in less than 50% of pharmacies (12), and, according to the American Pharmacists Association, some states reporting rising acute HBV infection rates also restrict the pharmacist’s authority to administer HBV vaccinations (eg, age limitations and prescription requirements) (13). For example, Maine, a state that has seen substantial increases in HBV infections, restricts pharmacist administration of the HBV vaccine to adults aged 18 years or older, and New York does not provide pharmacists with any authority to administer the HBV vaccine to their patients (13). Policy efforts should work to expand the pharmacist’s authority for administration of the HBV vaccine to address the gap in adult vaccination coverage across the United States.

The profession of pharmacy can look to existing best practices and successful models to provide vaccines having multiple doses or short follow-up requirements to identify methods for improving HBV vaccine services in the community pharmacy setting (12). These methods may include different reminder options, such as creating an order for follow-up doses when the first dose is administered, providing patient reminders through smartphone applications or text messaging, and aligning future vaccine dosages with medication synchronization models (12). Many pharmacy systems are accustomed to using reminder options such as these to improve adherence to chronic disease medications (12), and these same strategies can be leveraged to improve the community pharmacies’ ability to provide the full HBV vaccine series to their patients.

We must acknowledge that the rise of acute HBV infections is also associated with the opioid crisis, and that a portion of these acute infections will result in increased chronic HBV infections (approximately 10%) (14). Collaboration among public health agencies and pharmacy organizations, with particular emphasis on community pharmacies, should focus on designing and implementing new strategies for providing vaccines requiring multiple doses. This is an essential step to effectively mobilize pharmacists to increase the nation’s capacity to improve HBV immunization coverage among high-risk populations, including people who inject drugs. Furthermore, implementation of a successful model for providing multidose vaccines has the potential to positively impact population health far beyond opioid use disorder alone. Creating an effective model for providing the HBV vaccine series in the community pharmacy setting would likely translate to other preventable diseases requiring the administration of a multidose vaccine. The urgency to address the needs of people who inject drugs cannot be understated. Community pharmacists play a critical role in our nation’s multipronged approach to addressing the opioid crisis and must be mobilized to help prevent further increases in acute HBV infection rates across the country.
